# Multidrug-Resistant *Pseudomonas Aeruginosa* Bloodstream Infections: Analysis of Trends in Prevalence and Epidemiology

**DOI:** 10.3201/eid0802.010121

**Published:** 2002-02

**Authors:** Evelina Tacconelli, Mario Tumbarello, Silvia Bertagnolio, Rita Citton, Teresa Spanu, Giovanni Fadda, Roberto Cauda

**Affiliations:** Catholic University, Rome, Italy

**To the Editor:** Multidrug-resistant (MDR) *Pseudomonas aeruginosa* bloodstream infection has been described only in patients with cystic fibrosis (*1*) and in isolated outbreaks in intensive-care unit (ICU) or neoplastic patients (*2*–*4*). We investigated the percentage and clinical findings of patients with *P. aeruginosa* bacteremia having MDR strains in a 1,700-bed university hospital in Rome, Italy, over a 10-year period (1990-1999).

All consecutive patients with the first episode of community- or hospital-acquired *P. aeruginosa* bacteremia, according to the definition of the Centers for Disease Control and Prevention (*5*), were included in the analysis. The term MDR *P. aeruginosa* covered resistance to ciprofloxacin, ceftazidime, imipenem, gentamicin, and piperacillin. In patients with *P. aeruginosa* bacteremia, we evaluated age, gender, type of infection (hospital or community acquired), duration of hospitalization, risk factors, clinical findings, and outcome. Prognosis immediately before bacteremia developed was determined with the revised Acute Physiology and Chronic Health Evaluation (APACHE) III system (*6*).

Bacteria were identified by using API 20NE (Biomerieux, Marcy-l’Etoile, France). MICs were determined by broth microdilution in accordance with the methods of the National Committee for Clinical Laboratory Standards. Contingency data were analyzed by the two-tailed chi-square test or Fisher's exact test, and continuous data were analyzed by Student *t* test. Logistic regression analysis was used to determine which risk factors were independently significant. All statistical analysis was performed with the software program Statistics (Windows Systat Inc., Evanston, IL).

In the study period, *P. aeruginosa* was isolated from 358 of 379,190 hospitalized patients. Among 358 patients with *P. aeruginosa* bacteremia, 133 (37%) were hospitalized in medical wards, 103 (29%) in ICUs, 97 (27%) in surgical wards, and 25 (7%) in neonatology; 45 (12%) had HIV infection and 28 (8%) had hematologic malignancies.

For the study period, the overall hospital incidence of both nosocomial and community-acquired *P. aeruginosa* bacteremia was 0.94 per 1,000 hospital admissions. In particular, the incidence increased from 9.7 to 24.7 per 1,000 hospital admissions (p <0.01; chi square for trend) in ICUs. In HIV-infected patients, the incidence increased from 1.5 to 12.4 per 1,000 hospital admissions until 1996 when, after highly active antiretroviral therapy was introduced, it decreased to 0.7 (p = 0.01, chi square for trend).

The first case of MDR *P. aeruginosa* strain was isolated in the hematologic unit in 1992. After that, the hospital prevalence of MDR strains increased significantly (p=0.03) from 8% (3/37) in 1993 to 17% (9/54) in 1999. Overall, we observed 51 (14% of 358) cases of MDR *P. aeruginosa* bloodstream infections; 49 (96%) were nosocomial. The prevalence of MDR among the total *P. aeruginosa* bacteremia cases per ward was as follows: medical wards 1 (2%) of 60 (95% confidence intervals [CI] = 0.05-10); surgical wards 7 (7%) of 97 (95% CI = 3-15); hematologic ward 3 (11%) of 28 (95% CI = 2-28); ICUs 22 (21%) of 103 (95% CI = 13-31); and infectious diseases ward (in HIV-infected patients only) 18 (40%) of 45 (95% CI = 26-54).

The mean age ± standard deviation of patients with MDR *P. aeruginosa* infections was 52±12 years (range 29 to 77); 35 patients (69%) were men, and 9 (18%) were active intravenous drug abusers. The mean Apache III score at diagnosis of bacteremia was 41±17 (95% CI = 39-56). The mean concentration of circulating polymorphonuclear cells was 2,974±2,790/mm^3^ (95% CI = 2,181-3,796). In HIV-infected subjects, the mean number of peripheral CD4+ cells was 71±104 /mm^3^ (95% CI = 35-106). Advanced age (odds ratio [OR] = 1.07; 95% CI = 1.04-1.10, p<0.01), HIV infection (OR = 3.94; 95% CI = 1.10-14.11, p=0.03), intravenous drug abuse (OR=13.15; 95% CI=1.65-104.5; p=0.01), and previous therapy with quinolones (OR=3.21; 95% CI=2.14-23.33; p = 0.001) were independent risk factors on logistic regression analysis.

The overall mortality rate of patients with *P. aeruginosa* bacteremia was 31%; death rates were higher among patients with higher APACHE III score (mean 39 versus 27; p=0.01) and MDR *P. aeruginosa* infections (67% versus 23%; OR=15.13; 95% CI=1.90-323.13; p=0.001).

This prospective surveillance of *P. aeruginosa* bloodstream infections clearly indicates, for the first time, that multidrug resistance is statistically associated with HIV infection, as already observed for cystic fibrosis (*1*). We also identified a significant correlation between MDR *P. aeruginosa* bacteremia and intravenous drug abuse, advanced age, and previous quinolone use.

The association between isolation of MDR strains, HIV infection, and intravenous drug abuse (the most important HIV risk factor in Italy) is not an unexpected result. We have already demonstrated that hospitalized HIV-infected patients are at increased risk of acquiring nosocomial bloodstream infections compared with other immunocompromised hosts (*7*). Age is a well-known predisposing factor for bacterial infections. In particular, older HIV-infected patients progress more rapidly to AIDS (*8*).

Resistance following treatment with a single antimicrobial agent may be due in some circumstances to synergy between enhanced production of beta-lactamases and diminished outer membrane permeability (*9*). More emphasis is now given, however, to the energy-dependent efflux of antibiotics by *P. aeruginosa*. A single opening of a pump facilitates resistance to quinolones, beta-lactams, tetracycline, and chloramphenicol among the drug efflux (*9*). The recent characterization of a carbapenem-hydrolyzing metallo-beta-lactamase from *P. aeruginosa* opens new possibilities for reducing the spread of resistant strains (*10*).

One limitation of our study is the absence of genotypic analysis of MDR strains. However, we are confident that a general outbreak of MDR *P. aeruginosa* did not occur in our hospital. Nevertheless, limited outbreaks involving few patients in different wards remain a possibility. In summary, the observation that 14% of *P. aeruginosa* bloodstream infections are multidrug resistant is worrisome and reflects the growing worldwide problem of antimicrobial resistance. In particular, the fact that HIV-infected subjects are at increased risk, as are persons with cystic fibrosis, suggests the need for ongoing worldwide surveillance of *P. aeruginosa* in immunocompromised patients.
